# Congenital multi-organ malformations in a Holstein calf

**Published:** 2015-09-15

**Authors:** Rahim Hobbenaghi, Bahram Dalir-Naghadeh, Ali Nazarizadeh

**Affiliations:** 1 *Department of Pathobiology, Faculty of Veterinary Medicine, Urmia University, Urmia, Iran; *; 2 *Department of Internal Medicine and Clinical Pathology, Faculty of Veterinary Medicine, Urmia University, Urmia, Iran; *; 3 *Graduate Student, Faculty of Veterinary Medicine, Urmia University, Urmia, Iran.*

**Keywords:** Calf, Congenital malformation, Necropsy, Pulmonary hypoplasia

## Abstract

A 5-day-old female Holstein calf was necropsied because of lethargy, recumbency and anorexia. At necropsy, multiple gross defects were evident in several organs, including unclosed sutures of skull bones, asymmetrical orbits, doming of the skull bones, hydrocephalus, hydranencephaly, cleft palate, brachygnathia, ventricular septal defect, mitral valve dysplasia and rudimentary lungs. On microscopic examination, pulmonary hypoplasia was characterized by reduced number of alveoli, replacement of peri-bronchiolar smooth muscles with connective tissue and small masses of undeveloped cartilage around the small airways. The present report is the first description of the congenital pulmonary hypoplasia accompanied by numerous malformations in Holstein breed.

## Introduction

The major cause of economic loss in livestock production is newborn disease and congenital anomalies belong to this category. It has been estimated that 0.5 to 1.0% of calves are affected with congenital defects,^1^ which are structural or functional abnormalities and may or may not be obvious at birth.^2^ Numerous causes have been recognized for some defects , however, the etiology of most is still unknown.^3^ There is a wide range of possible defects, from a single structural change to the involvement of multiple organs or systems. Congenital defects may be genetic in origin or may be caused by environmental teratogens including toxic plants, drugs, viruses and physical agents (hyperthermia, irradiation).^2^

Congenital anomalies of the lung are rare and may reflect abnormal development of the lung bud, pulmonary circulation or both.^4^ The incidence of congenital pulmonary hypoplasia in human ranges from 9 to 11 per 10,000 live births with a very high fatality.^5^

Pulmonary hypoplasia is defined as reduced lung weight and usually reduced numbers of alveoli.^4^ This paper reports a case of congenital pulmonary hypoplasia associated with numerous other malformations in a Holstein calf. To the best of authors’ knowledge, this report represents the first description of the condition in Holstein breed.

## Case Description

On November 2013, a 5-day-old female Holstein calf was presented to the Veterinary Teaching Hospital of Urmia University for evaluation of the lateral recumbency, dullness, anorexia and immediate nasal regurgitation of milk following forced bottle-feeding. On physical examination, the calf appeared stupor, unable to stand or bear her weight on legs even with assistance and without suckling reflex. Rectal temperature was 38 ˚C. A load pansystolic cardiac murmur (grade 4/5) was auscultated on both sides of the thorax. Tachycardia, polypnea, prolonged capillary refilling time, cyanosis of mucous membrane and muzzle skin was also noticed. There was a big projection in posterior part of the cranium and a large cleft in hard palate. Laboratory analysis of the jugular blood sample revealed normal values for blood leukogram, total protein, minerals and electrolytes. However, polycythemia with PCV 58.0% was found in the complete blood count. 

The owner was recommended to seek further ancillary diagnostic test, however, because of the poor prognosis and financial limitations, he decided to euthanatize the calf. 

**Fig. 1:**
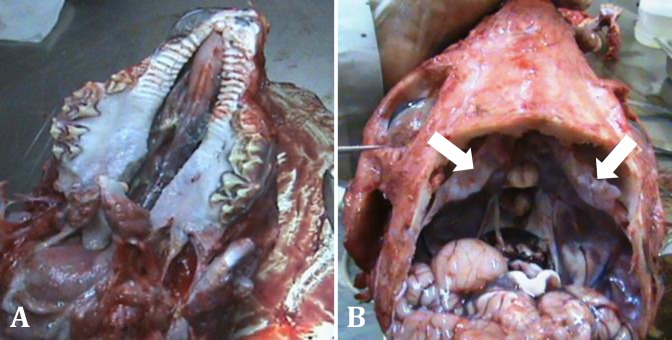
**A)** Palatoshcisis, a large cleft in hard and soft palate, nasal turbinate and nasopharynx have been exposed. **B)** Unclosed frontal sinus bones and the curvature of them to upward (arrows). Notice the asymmetric orbits.

**Fig. 2:**
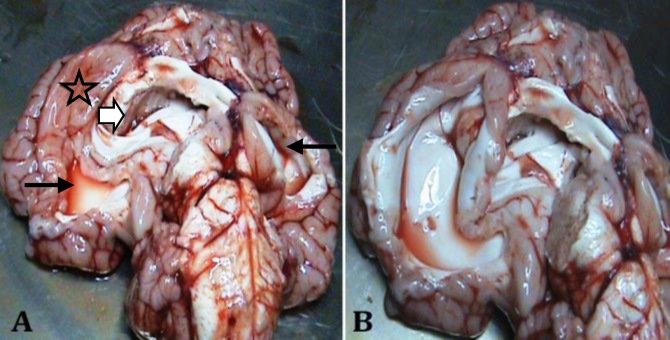
**A** **)** Ventral view of the brain shows hydranencephaly and hydrocephalus. Two big cavities in brain parenchyma (Thin arrows), partial lissencephaly (Asterisk) and the enlargement of lateral ventricles (Arrowhead). **B)** A large cystic cavity bounded by a thin rim of brain parenchyma

At gross necropsy, micrognathia of both mandible and maxilla (resulted in protruding of tongue from the mouth), dorso-caudal doming of the rear skull bones including frontal, occipital and parietal, asymmetry of the orbits and a large cleft palate extending entire the hard and soft palate were observed. Nasal and oral cavities were in extensive communication through the palatal defect resulting in some food materials lodged within the nasal cavities adjacent to the turbinates (Fig. 1A). The internal lamina of the frontal sinus was not fused with the occipital bone leading to bone defect in the both sides, communicating the brain with external lamina of the frontal sinus as well as with the temporal and occipital bones (Fig. 1B). This deformity provided a remarkable space around the brain, allowing cerebrospinal fluid (CSF) to leak into the sinus space through the opened defect. In addition two large fluid-filled cystic cavities bounded by a thin rim of brain tissue were found in the cerebral hemisphere. This finding is commonly described as hydranencephaly characterized by slightly smoother brain gyri. The enlarged lateral ventricles contained an abnormally large amount of CSF forming hydrocephalus (Fig. 2). 

The thoracic cavity contained slightly more fluid than the normal amounts. Heart dissection revealed a ventricular septal defect in the apical (trabecular) region. Right cusp of the mitral valve was distorted by a fibrotic plaque and the contralateral one was defective. The aorta and pulmonary trunks had their normal shapes and correct positions.

**Fig. 3:**
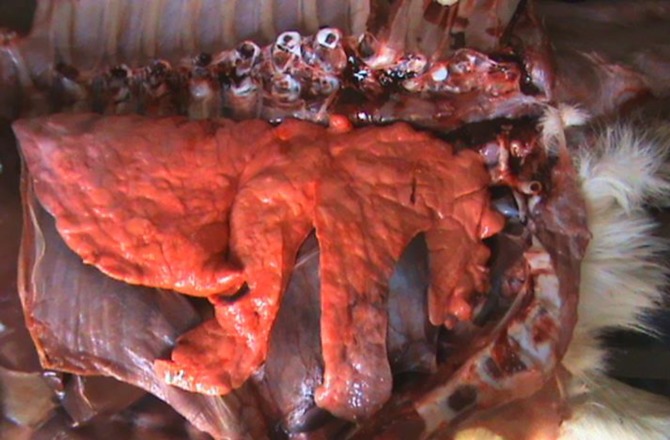
Right lateral view of the thoracic cavity, rudimentary lung with deep interlobar fissures (the ribs were removed).

The lungs were in their proper position within the thoracic cavity, however, they were rudimentary. There was no evidence of gross abnormality of the pleura, trachea and main bronchi. The right lung was smaller than the left one and the interlobar fissures were too deep (Fig. 3). The pleural surface and the cross section of the lungs were pink in color, however, the consistency was firmer than the normal lungs. Tissue sections at 5 µm were stained with hematoxylin and eosin (H & E) for histopathologic examination. 

**Fig. 4:**
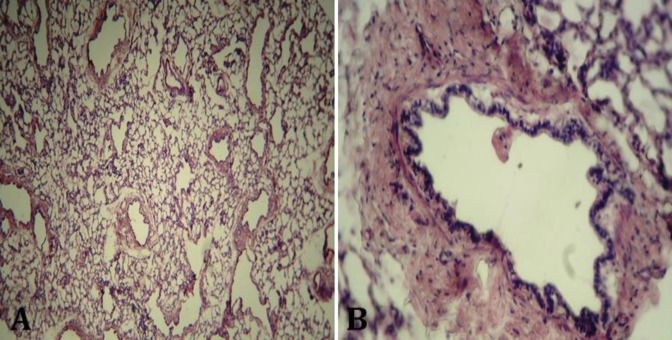
Histologic sections of hypoplasatic cranial lobe of the right lung. **A)** Notice numerous small airways in comparison with alveoli (H & E, 100×). **B)** A bronchiole has been surrounded by excessive connective tissue (H & E, 200×).

**Fig. 5:**
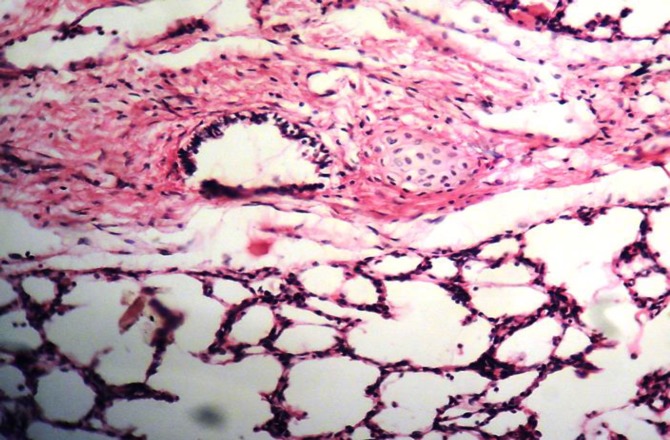
Photomicrograph of the lung, a disproportionate mass of undeveloped cartilage adjacent to the small airway (H & E, 200×).

At microscopic examination, the number of alveoli seemed a lot fewer than bronchiolar airways. The bronchioles had been covered by non-ciliated cuboidal epithelium. Peri-bronchiolar smooth muscle was mostly absent. Instead, layers of connective tissue had surrounded the bronchioles (Fig. 4). Occasionally, small masses of undeveloped cartilage existed adjacent to the small airways (Fig. 5). All of the alveoli were matured, containing one layer of squamous epithelia. Most of the alveoli were filled with a small amount of clear transudation occasionally containing red blood cells. Alveolar ducts, blood vessels and lymphatics were appeared normal.

Blood extravasations and hemorrhages were observed on the serosal surfaces, particularly on the epicardium and serosa of the intestine.

## Discussion

The paper describes a unique syndrome of congenital abnormalities in a newborn calf, affecting the respiratory, skeletal, neural and cardiovascular systems. 

Pulmonary hypoplasia was diagnosed based on the necropsy and light microscopic examination.^4^^,^^6^ Partly similar findings have been reported in Dexter and belted Galloway calves.^7^^,^^8^ In the present study, pulmonary hypoplasia was observed in association with several other congenital defects. Because of interdependent development of tissues and organs, it is not uncommon for more than one anomaly to appear in an animal.^9^ The lung was rudimentary and the number of alveoli was a lot fewer than the small airways. These gross and microscopic findings are consistent with occurrence of hypoplastic process in the pulmonary parenchyma.

Cyanosis in this case may be attributable to the pulmonary hypoplasia and interventricular septal defect. Such defect usually results in left to right shunt with no cyanosis and no impact on arterial oxygen tension. However, pulmonary over-circulation over time can lead to progressive pulmonary hypertension, eventually halting and then reversing the shunt direction. In addition, pulmonary hypertension as a complication of pulmonary hypoplasia can be occurred.^10^ Mitral valve lesion may be a further reason of pulmonary hypertension in this case. 

The polycythemia of the calf described in the present report might be secondary and can be related to hypoxia and right-to-left shunting ventricular septal defect. The origin of serosal surface hemorrhages and fluid accumulation in the alveolar space can be ascribed to general hypoxia and circulatory disturbances, respectively.

In human medicine, pulmonary hypoplasia is considered a rare congenital anomaly arising from embryological defect or secondary to some other malformations. In particular, pulmonary hypoplasia is tightly correlated with congenital diaphragmatic hernia. Furthermore, about one third of cases have cardiovascular malformations and lesser proportions have skeletal, neural, genitourinary, gastrointestinal or other defects.^11^ Based on the literature, pulmonary hypoplasia is arisen by conditions that compress the lung, such as congenital diaphragmatic hernia, intrathoracic masses and pleural effusion.^4^ However, diaphragmatic hernia is uncommon in farm animals.^12^ In the present case, we did not find any evidence to conclude that the lung had been compressed within the thoracic cavity.

Neurocranial defects observed in this case are considerably similar to the lesions that caused by Akabane virus, an epizootic teratogen virus and vitamin A deficiency. However, hypoplasia of the lung is rare and mainly occurs in lambs, infected by Akabane virus.^13^ Moreover, malformations incurred by vitamin A deficiency are mostly limited to ocular and cranial lesions in cattle and pulmonary hypoplasia is reported in piglets suffering from maternal deficiency of vitamin A.^3^^,^^4^^,^^14^^,^^15^

Less common than the aforementioned virus, intra-uterine infection with Schmallenberg (a novel orthobunyavirus) and bovine viral diarrhea virus can lead to hypoplasia of the lungs and neurocranial defects.^16^^,^^17^

In this report, we described occurrence of pulmonary hypoplasia in a Holstein calf affected with multiple congenital defects. Decreased number of alveoli, replacement of peri-bronchiolar smooth muscles with layers of connective tissue and hypoplastic cartilage masses around the small airways were the remarkable findings at the histopathologic examination.
